# Sport, doping and female fertility

**DOI:** 10.1186/s12958-018-0437-8

**Published:** 2018-11-19

**Authors:** Sandro La Vignera, Rosita A. Condorelli, Rossella Cannarella, Ylenia Duca, Aldo E. Calogero

**Affiliations:** Department of Clinical and Experimental Medicine, University of Catania, Policlinico “G. Rodolico”, via S. Sofia 78, 95123 Catania, Italy

## Abstract

This article is a review that addresses the following topics, divided by paragraphs. The first paragraph investigates the effects of physical activity on ovarian function, analyzing in particular the changes concerning the serum concentrations of follicle-stimulating hormone, luteinizing hormone, prolactin, growth hormone, thyroid hormones, leptin, ghrelin, neuropeptide Y. The second paragraph analyzes the effects of doping on the hypothalamic-pituitary-ovarian axis. Finally, the last paragraph analyzes the PCOS category, evaluating the effects of hyperandrogenism in relation to athletic performance.

## Introduction

The repercussions that physical exercise has on ovarian function represent a controversial aspect and not frequently evaluated in the clinical practice. The variables are many and may relate to the characteristics of physical activity (aerobic or anaerobic, agonistic or non-competitive, duration of training sessions, frequency of weekly sessions), or the characteristics of the woman (age, menstrual cycle regularity, body weight, diet, possible presence of PCOS, pregnancy research). A separate aspect concerns the possible reflexes of hyperandrogenism of women with polycystic ovary syndrome (defined as a syndrome of ovarian dysfunction along with the cardinal features hyperandrogenism and polycystic ovary morphology [1]) on their athletic performance.

### Physical exercise and ovarian function

Menstrual irregularities occur among high-intensity-exercising women [2]. The prevalence of functional hypothalamic amenorrhea has been reported as high as 40% and that of oligo-amenorrhea ranges from 9 to 40% in athletes. This prevalence is higher than that found in non-athletic women (5–11%) [2]. Similarly, anovulation and luteal phase deficiency are more likely to occur among exercising compared to sedentary women [2].

The “critical fat” hypothesis has been postulated more than 45 years ago by the epidemiologist Rose Frisch, who proposed that a critical amount of fat is necessary either for the onset of puberty and for the preservation of reproductive function. Indeed, adipokines from adipose tissue sensitize the hypothalamic-pituitary-ovarian (HPO) axis providing a signal for the onset of puberty and for its function [3].

Although such a hypothesis well explains the reason why obese teenagers experience menarche earlier than thinner peers, it is not applicable in a number of situations, such as lean girls experiencing the menarche before achieving a critical fat mass, or in the case of un-uniform experience of irregular menses after critical weight loss or extreme exercise [4].

In this regard, the “metabolic fuel” hypothesis has been postulated, assigning to the energy availability per se a role in the regulation of the HPO axis function. According to this hypothesis, the negative energetic balance, more than the fat mass content, would be responsible for reproductive dysfunction in exercising women [4]. In deep detail, an energy availability below 30 kcal/Kg/lean body mass [LBM]/day has become the best explanation for exercise-induced reproductive disturbances, especially in lean athletes [5–7]. The negative energy balance would stimulate compensatory mechanisms, which in turn translates into HPO axis suppression [8].

A number of studies evaluated the HPO axis (gonadotropins, prolactin, 17β-estradiol) in the early follicular phase of eu-, oligo- and amenorrheic exercising women and healthy controls. The main findings are discussed below.

#### Luteinizing hormone, follicle-stimulating hormone and prolactin

Exercise can impair luteinizing hormone (LH) secretion in sedentary women. Indeed, in a cohort of sedentary young regularly menstruating women, an impaired LH pulsatility has been observed after aerobic exercise in case of negative energy balance (< 30 kcal/Kg/LBM) [5]. Similarly, lower LH levels compared to those at baseline in early and late follicular and luteal phases were described in 25 young, sedentary and regularly menstruating women after a 90-min physical exercise on a motor driven treadmill at 55–60% of maximal oxygen uptake [9]. On the contrary, serum LH levels measured in the follicular phase do not seem to differ among amenorrheic exercising women, cycling exercising women and cycling sedentary women. In detail, women were asked to cycle at a workload of 200 Kg*m/min (corresponding to 32.69 watts), which was increased to 200 Kg*m/min every 2 min until exhaustion [10]. In contrast to these findings, Laughlin & Yen (1996) reported 30 and 50% decrease in LH pulse frequency respectively in cycling and amenorrheic athletes compared to sedentary cycling women [11].

Follicle-stimulating hormone (FSH) serum levels measured in the follicular phase have been reported to be lower compared to those at baseline after aerobic exercise in sedentary women [9], whereas no difference has been found in exercising compared to sedentary woman [10, 11].

Contrasting data have been reported on serum prolactin (PRL) levels. In a case-control study on 20 women (among them 5 were non running women, 5 eumenorrheic, 4 oligomenorrheic, 6 amenorrheic runners), a higher rise in PRL levels was found in the exercising women compared to sedentary ones after aerobic exercise [10]. On the contrary, amenorrheic exercising women showed lower PRL levels compared to both cycling exercising and cycling sedentary women [11].

#### Growth hormone

Excessive exercise seems to impair growth hormone (GH) secretion. Indeed, a higher rise in GH levels has been reported in exercising women compared to non-running women after aerobic exercise [10]. Furthermore, an irregular GH pulsatility was described in amenorrheic compared to cycling exercising women [12] and an accelerated pulse frequency, both being responsible for a 70–80% augmentation of 24 h GH concentration in amenorrheic and cycling exercising women compared to cycling sedentary controls [11].

#### Thyroid hormones

In exercising athletes experiencing irregular menstruation and HPO axis function abnormalities, a hypothalamic-pituitary thyroid axis impairment seems to occur. In fact, despite thyroid stimulating hormone levels did not differ, free-triiodothyronine and free-thyroxin were lower in amenorrheic athletes compared to cycling exercising and sedentary women [13]. In addition, total T3 levels were lower also in amenorrheic exercising women compared to cycling sedentary, cycling exercising and anovulatory exercising women; furthermore, total T3 levels were lower both in cycling and anovulatory exercising women compared to cycling sedentary controls [14]. Similar results have been reported also elsewhere [15]. Low total T3 levels positively correlate with the lower resting energy expenditure/fat free mass ratio in exercising groups with irregular menstruation compared to sedentary cycling women [15]. At the light of such findings, the decrease of T3 levels might represent a compensatory mechanism in case of negative energy balance, to reduce calories consumption.

#### Leptin, ghrelin, neuropeptide Y

Leptin, ghrelin, neuropeptide Y (NPY) may be defined as detectors of the metabolic status.

Leptin is a 16 kDa peptide secreted by the adipose tissue, whose production is stimulated by food intake. This peptide sensitizes the HPO axis and its deficiency results in infertility both humans and rodents, due to HPO axis deficiency. Leptin receptors have been identified in the hypothalamus, in the anterior pituitary and in the ovary [8]. In-vivo studies performed in humans reported a mild improvement of hypothalamic amenorrhea after treatment with recombinant leptin [16]. Studies performed in physically active women observed lower leptin levels in all exercising groups compared to the sedentary one [11, 14]; in addition, lower leptin levels have been reported among amenorrheic compared to cycling exercising women [17]. Hence, leptin levels may represent a metabolic signal, which provides a link between adipose tissue, energy availability and the HPO axis [17].

Ghrelin is a 28 aminoacid peptide that is synthetized in response to negative energy balance. Its receptors have been identified in the hypothalamus and their activation stimulates food intake and limits energy expenditure [3]. Little is known about the role of this peptide on human HPO axis. According to in-vitro studies, central ghrelin administration inhibits gonadotropin-releasing hormone (GnRH) and LH secretion [18, 19]. Interestingly, higher ghrelin levels have been reported in amenorrheic exercising women compared with both the other exercising non amenorrheic groups and with cycling sedentary controls [14, 15], thus confirming the inhibitory role of ghrelin in the function of the HPO axis.

NPY seems to exert an inhibitory action upon the HPO axis [20–23]. Its receptors have been identified within the arcuate nucleus [3] and its release is stimulated by ghrelin [24]. Higher NPY levels have been recorded in underweight amenorrheic women [25, 26]. No study evaluated its levels in exercising women so far.

These findings are summarized in Table 1. The main bias of the reported studies regards their heterogeneity. Indeed, information and/or outcomes such as daily energy expenditure and calories intake, together with women’s lean and fat mass has not been reported everywhere, thus limiting the studies comparability.

**Table 1:** Hormonal findings in exercising and sedentary women

Population	Hormonal findings	References
Sedentary women after aerobic exercise vs. baseline	Impaired LH pulsatility ↓LH, ↓FSH	[5, 9]
Exercising vs. sedentary women	Impaired LH pulsatility N LH, N FSH, ↓↑PRL, ↓FT3, ↓FT4 ↑GH, ↑ghrelin, ↓leptin	[10, 11, 14, 15]
Amenorrheic vs. cycling exercising women	Impaired LH pulsatility ↓PRL ↓FT3, ↓FT4 ↑GH, ↑ghrelin	[10–15]

*Abbreviations*: *FT3* free-triiodothyronine, *FT4* free-thyroxin, *FSH* follicle-stimulating hormone, *GH* growth hormone, *LH* luteinizing hormone, *N* non-significantly different, *PRL* prolactin

#### Fertility

Evidence suggests that regular physical activity positively affects female fertility and the offspring health, although this effect seems to depend on the exercise intensity [27]. An observational cohort study performed on 41 obese infertile women on regular physical activity (cases) and 175 obese infertile controls undergoing to in-vitro fertilization reported a 3-fold higher likehood for clinical pregnancies and live births in cases compared to controls [28]. Therefore, irrespective for the body weight loss, physical exercise seems to display beneficial effects on human pregnancy. The authors speculated that this might be due to a differential exercise-induced expression of endometrial proteins involved in its receptivity [28]. Another study reported higher pregnancy rates among women having more active lifestyles the year before in-vitro fertilization compared to sedentary ones [29]. Interestingly, voluntary exercise seems to improve oocyte quality in obese murine model [30]. In detail, it increased oocytes β-oxidation enzyme hydroxyacyl-coenzyme A dehydrogenase levels in mice which have been fed with a high-fat diet, thus reversing lipid accumulation in germinal vesicle stage oocyte [30]. Previous studies indicated that a dietary intervention generally fails to achieve such oocyte quality improvement [31].

Accordingly, the positive effects of exercise on fertility in obese female rats have already been described. In these rats, exercise, in absence of weight loss and performed before and during pregnancy, seems also to exert beneficial effect on the offspring metabolism (lower glucose, leptin and triglycerides serum levels in the offspring of rats undergone to exercise compared to those of the offspring of not exercising rats) [32]. Interestingly, an ongoing randomized controlled trial is evaluating the effects of regular moderate-intensity exercise in human offspring health (Trial registration number: ACTRN12612000932864) [33].

Despite such evidence, it should be kept in mind that high intensity physical activity has a negative effect on human female fertility. A population-based health survey on 3887 women found that increased frequency, duration and intensity of exercise were associated with increased subfertility. Exercising with exhaustion was associated with a 2-fold higher risk of fertility problems compared to low intensity exercise [27]. Therefore, moderate-intensity exercise might be suggested to improve female fertility.

### Effects of doping on ovarian function

Appearance- and performance-enhancing drugs (APEDs) are substances of different chemical nature used by athletes, amateur sportsmen and body-builders to improve sports performance or physical appearance. They include both legal dietary supplements and illicit pharmacologic agents [34]. Every pharmacologic agent used as APEDs may cause negative side effects involving different organs and systems, including the reproductive one.

Among APEDs, the drugs most used all over the world and the one capable of causing the greatest damage to the reproductive function are anabolic-androgenic steroids (AASs) [35]. Other substances used less frequently, and often in association with AASs, are GH, insulin-like growth factor 1, insulin, erythropoietin, stimulants, diuretics, levothyroxine, and gamma-hydroxybutyrate [35].

AASs are a group of synthetic derivatives of testosterone (T) with anabolic and masculinizing effects. There are four major classes of AASs (oral, injectable oil-based, injectable water-based, transdermal gel) and at least 30 anabolic-androgenic steroid compounds [36] (Table 2). According to a recent meta-analysis, the lifetime prevalence rate of their use in women is 1.6% [37]. Among AASs, women prefer most frequently oral oxandrolone because it is considered less androgenic than the T esters [38]. Other commonly abused steroid supplements include precursors of T, such as androstenedione and dehydroepiandrosterone (DHEA) (Table 2). Women use these last two more frequently because they cause a greater increase in T in the female subjects than in men [36].

**Table 2:** List of the main anabolic androgenic steroids used as doping

Testosterone (transdermal)		
17α-alkyl derivates (oral)	Ethylestrenol Methandienone Methyltestosterone Oxandrolone Oxymetholone Stanozolol	
17β-ester derivates (parenteral)	Boldenone Drostanolone Methenolone Nandrolone Testosterone esters	Testosterone cypionate Testosterone enanthate Testosterone propionate Testosterone undecanoate Others
Testosterone precursors	Androstenedione Dehydroepiandrosterone (DHEA)	

As well as T, AASs penetrate inside the cells and bind to the cytoplasmic androgen receptor. The androgen-receptor complex, through the binding with DNA sequences called androgen response elements, activates the transcription of mRNA responsible for the increased synthesis of several proteins, including actin and myosin in skeletal muscles [36]. Moreover, AASs act as glucocorticoid antagonists, so their anabolic effects also depend on the inhibition of muscular catabolism induced by glucocorticoid during physical stress [39]. Finally, some authors suggest other mechanisms for the ergogenic effect of AASs: psychotropic actions; down-regulation of myostatin; induction of human growth hormone and insulin-like growth factor 1 synthesis, erythropoiesis stimulation [39].

In female athletes, clitoromegaly and menstrual alterations (delayed menarche, oligomenorrhea, secondary amenorrhea, dysmenorrhea and anovulation) are the main side effects reported during AASs use [40].

#### Effects on the hypothalamic-pituitary-ovarian axis

Gonadal function depends on the presence of intact hypothalamic-pituitary-gonadal axis activity, involving pulsatile secretion of the GnRH by the arcuate nucleus of the hypothalamus, and of gonadotropins (LH and FSH) by the pituitary gland [40].

A recent systematic review and meta-analysis revealed that long-term AASs use results in prolonged hypogonadotropic hypogonadism in both sexes. In almost all studies included in the meta-analysis, there were decreased serum LH and FSH levels during AASs use [40]. AASs suppress gonadotropin release from the pituitary gland by a negative feedback mechanism, either directly on the pituitary gland or indirectly by suppressing the hypothalamic GnRH release. This results in a down-regulation of both gonadotropins and a decreased secretion of endogenous steroids [36–40].

Secondary amenorrhea with anovulation is a reversible effect caused by AASs, even if complete recovery of the axis can take weeks or months after suspension of AASs use [41]. However, since strenuous exercise can contribute to a state of hypogonadotropic hypogonadism, in the absence of controlled studies, disentangle the effects of sports from those induced by AASs is very difficult [42–44].

#### Effects on secondary sexual characters and integumentary apparatus

Adverse effects in women following chronic AASs use include masculinization (clitoris hypertrophy, male pattern baldness and hirsutism), acne, oily skin, and breast atrophy. The virilizing effects of AASs use by women are similar to the clinical features of the virilizing syndrome associated with congenital adrenal hyperplasia and adrenal carcinoma [36].

Hirsutism and alopecia are frequent and their degree depends on dose and duration of AASs abuse. Also laryngeal tissue has androgen receptors, so deepening of the voice is part of the virilisation that androgenic substances and AASs can cause in women. Lowering of the voice is caused by growth of the larynx in girls and by thickening of the vocal chords in women and is often accompanied by hoarseness [41].

Cutaneous modifications, hirsutism, alopecia and reduction of breast size are reversible side effects, while clitoris hypertrophy and deepening of the voice are possibly irreversible side effects of AASs use in women, but no well-documented case reports or studies are available [41].

#### Effects on breast and endometrial carcinogenesis

Data on the association between AASs abuse and breast cancer are controversial. In the absence of controlled studies, scientific evidences mainly derive from observations of women with polycystic ovarian syndrome (PCOS) and of woman treated with low-dose testosterone for female sexual dysfunction.

In premenopausal women most studies do not demonstrate an association between T levels and breast cancer [45]. According to this, women with PCOS, a syndrome characterized by androgen excess, do not show an increased risk of breast cancer [46].

In postmenopausal women the evidences are less clear. Some studies showed no significant association between breast cancer risk and endogenous androgens [47, 48]; while other studies showed association between circulating androgens levels (T, free T, androstenedione, DHEA, DHEAS) and postmenopausal breast cancer [49–55].

In postmenopausal treated woman, therapy with only androgens appear safer than combined treatment with estrogens plus testosterone [45]. Some studies even show that testosterone therapy in postmenopausal women reduces the incidence of breast cancer [56, 57]. Effectively, testosterone in vitro blocks breast cells proliferation and the expression of estrogen receptor genes, with an antiproliferative and proapoptotic action, probably mediated by the androgen receptor. But, in vivo, most of exogenous androgens are partially metabolized in breast tissue to estrogens, so further investigations are required [41].

Similarly, at endometrial level, therapy with both estrogen and T in postmenopausal women seems to promote endometrial hyperplasia and polyps formation, probably due to T-to-estradiol conversion by aromatase activity and reaching of elevated endometrial estrogen levels [58]. On the contrary, T given without concomitant estrogen promotes endometrial atrophy [59]. Therapy with DHEA in postmenopausal woman seems have no endometrial effects [60].

In conclusion, we can argue that in female AAS abusers, belonging in most cases to the category of women in premenopausal age, the use of AASs cannot be causal for breast and endometrial cancer. More attention should be paid to patients taking at the same time estrogen and AASs, but there are no studies on the subject.

These findings are summarized in Table 3.

**Table 3:** Findings in AASs users

Findings	References
*Hormonal findings:*	[36–40]
↓LH	
↓FSH	
↓GnRH	
↓Endogen steroids	
*Integumentary apparatus:*	[41]
Hirsutism	
Alopecia	
Acne	
*Signs of virilization:*	[41]
Reduced breast size	
Lowering of voice	
Clitoris hypertrophy	

*Abbreviations*: *AAS* anabolic-androgenic steroids, *FSH* follicle-stimulating hormone, *GnRH* gonadotropin releasing hormone, *LH* luteinizing hormone

### Is PCOS a “doping” condition?

In some athletes with menstrual disorders, in particular swimmers [61, 62] and endurance athletes [63], another endocrine status characterized by mild hyperandrogenism has been described. Rickenlund and colleagues reported that T, LH, and PRL correlate positively and cortisol negatively with the number of menstruations per year and that hyperandrogenism is more frequent in oligomenorrheic than in amenorrheic athletes. Most of hyperandrogenic athletes had also typical picture of polycystic ovaries on ultrasound [64]. They concluded that oligomenorrhea and amenonorrhea may be symptoms of two distinct and hormonally different conditions: one - functional hypothalamic amenorrhea - acquired and resulting from insufficient dietary intake or strenuous exercise; the other one - hyperandrogenic oligomenorrhea/polycystic ovary syndrome (PCOS) – probably primitive [64].

Hypothetically, hyperandrogenism may imply competitive advantages and could play a role in the selection of subjects to sport activities. This could explain the higher prevalence of hyperandrogenism and PCOS in athletes compared to the general population [65]. According to the Rotterdam consensus, PCOS is diagnosed when at least two of the three following signs are present: 1) oligo- or anovulation, 2) clinical and/or biochemical signs of hyperandrogenism, and/or 3) polycystic aspect of the ovaries at the ultrasound examination [1].

Following, we evaluated all the available data concerning the occurring of hyperandrogenism and PCOS among different kinds of athletes and their role in the athletic performance. Therapeutic strategies of PCOS include treatment of metabolic disorders (e.g. hyperinsulinemia, insulin-resistance) with insulin sensitizers and/or physical activity, treatment of hirsutism and/or other clinical signs due to hyperandrogenism with antiandrogens and menstrual irregularities with hormonal contraception [66]. The possible interference of such treatments in athletic performance has not been evaluated so far.

Swedish female Olympic athletes not using hormonal contraception have a prevalence of 27% of menstrual disturbances, mainly oligomenorrhea. Menstrual alterations are frequent in endurance athletes and, contrary to what is believed, the most common endocrine abnormality is not hypothalamic suppression, but PCOS [65]. Ultrasound evidence of polycystic ovaries was found in a higher percentage (37%) of athletes not using hormonal contraception, particularly in power athletes, compared to the estimated prevalence (20%) in the general population [67]. Athletes with PCOS showed higher T concentration and free androgen index than regularly menstruating or non-PCOS Olympic athletes [65].

In adolescent competitive swimmers, a high prevalence of hyperandrogenism has been shown [62]. Over 60% had T level > 0.5 ng/mL, a serum T cutoff that in adolescents is considered the upper limit; 50% had menstrual disorders and about 45% presented the Rotterdam criteria for PCOS. The authors hypothesized that hyperandrogenism may have preceded the intensive training, predisposing the girls to choice a sport - such as swimming - where muscular strength is needed. Authors also speculate that intensive training may have attenuated the clinical expression of hyperandrogenism [62]. In fact, the positive effect of moderate-intensity exercise on PCOS is well known to the point that exercise is considered, together with a mild reduction of body weight, the first-line therapy in PCOS [68].

Bermon and colleagues measured serum androgen levels of 849 female athletes from 163 countries taking part in the 2011 IAAF World Championships in Daegu (South Korea) to establish normative serum androgen values for elite female athletes and to estimate the occurrence of hyperandrogenism among this population [69]. They found that median T and free-testosterone (fT) values were close to those reported in sedentary young women with a 99th percentile T level of 3.08 nmol/L. No significant difference was found between the ethnic groups. Throwers, sprinters, and jumpers (power disciplines) showed higher levels of androgens than long-distance runners did. They also showed a prevalence of hyperandrogenic 46,XY disorder of sex development (7 per 1000), 140 times higher than in the general population. This was envisioned as an indirect evidence for performance-enhancing effects of high T concentrations in female athletes [69].

However, excluding subjects with hyperandrogenic disorder of sex development who are exposed to high levels of androgens from prenatal age, since the athletes often begin training before menarche, the influence of intensive training on pubertal development and menstrual function cannot be excluded. Female athletes with oligomenorrhea and hyperandrogenism show a higher frequency of delayed puberty [64, 70]. Therefore, some authors hypothesized that hyperandrogenism may be a consequence of intensive training rather than a primitive factor influencing sports performance and, consequently, selection [70].

Łagowska and Kapczuk evaluated the hormonal status of a sample of Polish dancers and athletes with menstrual disorders. All subjects had a negative energy balance with energy availabilities <30 kcal/kg fat free mass/day. They were divided into three groups depending on T levels: low, normal and high. High T levels were more frequent in ballet dancers than in athletes (85.7% vs. 29%), in girls who began training earlier, and in girls whose training period was longer. Despite T levels, none of the subjects in the high T group had clinical signs of hyperandrogenism (hirsutism, acne, alopecia, voice deepening). The authors excluded in all hyperandrogenic subjects the main conditions that can cause hyperandrogenism (PCOS, congenital adrenal hyperplasia, Cushing’s syndrome and androgen-secreting tumours). Interestingly, the high T group showed the lowest energy and carbohydrate intake and the lowest energy availability [70]. Therefore, the authors hypothesize that the increase in T levels could represent a sort of protective mechanism against excessive weight loss thanks to T property of stimulating the growth of lean tissue mass. Furthermore, among dancers hyperandrogenism can be considered a useful adaptive reaction, since it can reduce the risk of bone fractures [70]. This is in agreement with other findings showing that hyperandrogenic female athletes with menstrual disorders have an anabolic body composition with higher values of bone mineral density (BMD) and LBM compared to normoandrogenic athletes [63].

The increase in T levels resulting from a chronic negative energy balance in female athletes in endurance sports may, in turn, perturb the hypothalamic-gonadotropin axis and lead to PCOS in the long term. Indeed, polycystic ovaries are considered a result of a combination of long-standing hyperandrogenism and anovulation, regardless of origin [63].

Several authors have wondered whether hyperandrogenism in athletes, regardless of its etiology (primitive or secondary to intensive training), may influence the physical fitness and could entail an advantage in physical performance. Rickenlund and colleagues compared the physical performance of sedentary controls and endurance athletes. The latter were divided into three groups: hyperandrogenic oligomenorrheic/amenorrheic (H-OAM), normoandrogenic oligomenorrheic/amenorrheic (N-OAM), and regularly menstruating (RM) athletes. Maximal oxygen uptake and pulmonary ventilation were measured while the subjects ran on a motor-driven treadmill and endurance was evaluated using the Beep test, a multistage progressive shuttle-run test. The results showed that H-OAM performed better than the other athlete groups, reaching a higher final level in the Beep test and a significantly higher VO_2_ max during the treadmill exhaustion test. H-OAM showed higher lactate concentrations than N-OAM or RM, probably because they ran on the treadmill for a longer time and did better on the Beep test. Finally, all athletes showed significantly higher isometric leg strength than sedentary controls, but the numerically highest mean value was found in H-OAM [63]. These data suggest that mild hyperandrogenism may improve performance among endurance athletes. However, interestingly, there were no differences in handgrip muscle strength between the groups, indicating that H-OAM performed better because of training and not because of their hyperandrogenic condition as such [63]. Therefore, hyperandrogenism could indirectly improve physical performance enhancing the ability to withstand high training loads.

In 2006, Cardinale and Stone established the relationship between T levels and vertical jumping ability in a cohort of elite athletes, 22 women and 48 men [71]. Among female athletes, there were 12 sprinters and 10 volleyball players. Authors found a significant positive relationship between T levels and vertical jump performance. Furthermore, when the two group of female athletes were compared, T levels and vertical jumping ability resulted significantly higher in sprinters than in volleyball players [71]. These results indicate that T positively influences explosive performance and that different types of sports and/or training may have a different influence on hormonal levels.

Cook and colleagues compared the baseline hormonal levels of eighteen elite and non-elite female athletes over a 12-week period. Athletes came from track and field, netball, cycling, swimming, and bob skeleton, had regular menstrual cycling and were not on hormonal-based contraception [72]. The elites (n. 9) were international and non-elites (n. 9) were national level competitors, and both groups were matched by sport. Author found that fT concentrations of the elite athletes were more than double than those of non-elites athletes (87 vs. 41 pg/ml). Free cortisol concentrations were also greater in the elite group than the non-elites (2.90 vs. 2.32 ng/ml). They concluded that higher fT concentrations could produce a better physical performance at higher work rates, such as the ones requested in elite sport. They also speculate that higher T levels could confer an advantage to female elite athletes influencing their behavior in term of greater dominance and competitiveness [72].

To test the influence of serum androgen levels on performance, Bermon and Garnier classified female elite athletes in tertiles according to their fT concentration and compared the best competition results achieved in the highest and lowest fT tertiles. Subjects were athletes taking part in the 2011 and 2013 IAAF World Championships and belonging to the following discipline categories: throwing, jumping, sprinting, heptathlon, middle distance running, long distance running, and race walking. A total of 1332 competition performances were recorded [73]. The type of athletic event did not influence fT concentration among elite women but female endurance runners showed decreased androstenedione and DHEA sulphate concentrations when compared with other athletes. Authors found that female athletes with the highest fT tertile performed significantly better in 400 m, 400 m hurdles, 800 m, hammer throw, and pole vault. In consideration that androgens are erythropoietic hormones and because in sprinting and middle distance running events athletes with the highest fT levels showed also higher hemoglobin concentrations, authors postulate that better results in these disciplines could be partially explained by the increase in the oxygen-carrying capacity and (non-bicarbonate) extracellular buffering capacity.

Hammer throw and pole vault are disciplines that require a high level of power and strength but also great spatial abilities. Sex differences in spatial abilities are well documented and males perform better than females in the mental rotation task [74]. Therefore, Authors speculate that androgens in some sportswomen could improve performance modulating visuospatial neural activity [73].

Recently, Eklund and colleagues examined serum androgen profile in relation to body composition and physical performance of 106 women Swedish Olympic athletes, belonging to three different sport categories: power, endurance and technical. Authors compared endocrine variables and androgen metabolites between these three groups and with a group of 117 sedentary controls. The athletes demonstrated significantly higher levels of the precursor androgens DHEA and 5-androstene-3β,17β-diol and the metabolite etiocholanolone glucuronide, significantly lower levels of estrone, higher bone mineral density and more lean mass compared with controls [75]. The frequency of menstrual disorders was higher among the athletes and the athletes with menstrual disorders had higher serum levels of etiocholanolone glucuronide than the other athletes. Significant positive correlation was found between androgen levels and total BMD and Z-score in all groups. Among the three groups of athletes, power athletes demonstrated the highest BMD and Z-score, and endurance athletes had the highest values of lean body mass. Explosive performance in the athletes was evaluated through two tests - squat jump and countermovement jump - and resulted significantly correlated with serum levels of DHEA, 5-androstene-3β,17β-diol and dihydrotestosterone supporting a role of endogenous androgens for athletic performance in women athletes [75].

Although poor, literature data overall indicate that female athletes with high androgen levels (either from endogenous or exogenous origin) have a competitive benefit of 2–5% over those with androgen levels within the normal female range [76]. The advantage would seem to be greater in explosive performance and in those disciplines that require high visuo-spatial abilities. In fact, androgens act not only on the muscles, increasing protein synthesis and lean body mass percentage, but also on the oxygen transport and in the modulation of the visuo-spatial cerebral activity. Furthermore, T has behavioral effects: by increasing aggression, dominance and risk taking it could also enhance competitiveness and influence the choice of the sport and the performance [72, 73]. The most frequent cause of mild hyperandrogenism is PCOS, which has a higher prevalence among the athletes than in the general population [62, 65]. Therefore, speculatively, we can assume that hyperandrogenic girls with PCOS could have a slight advantage compared to normoandrogenic athletes in disciplines requiring muscular strength, such as swimming and sprinting, in middle distance running and in disciplines requiring high visuo-spatial abilities, such as gymnastics, hammer throw, and pole vault. Consequently, they could be able to reach higher levels in the aforementioned sports.

However, some authors do not exclude the possibility that hyperandrogenism may be induced by an intensive training started before puberty and that hyperandrogenism could, in long term, result in a PCOS-like syndrome [63, 70]. In fact, hyperandrogenism could represent an adaptive response of the organism aimed at counteracting the catabolic state induced by an intensive training with negative energy balance. The latter hypothesis could be indirectly supported by the experimental evidence that female rats with PCOS induced by prenatal androgen exposure, show reduced voluntary running. In fact, normal mice voluntarily ran several kilometers per day, while mice with PCOS ran approximately one-third less distance [77]. The mechanisms underlying reduced running does not seem to be related to decreased exercise capacity but is more likely due to decreased reward from running. Thus, women with PCOS may be “lazier” and less inclined to undertake sports activities at high levels, but this hypothesis requires further investigations.

## Conclusions

The prescription of regular physical activity by the endocrinologist represent an important step of the clinical evaluation, in relation to different aspects. In the male it has been widely documented that aerobic physical activity reduces the insulin resistance associated with hypogonadism [78] and improves the quality of erectile function [79, 80]. In women, it is appropriate to consider the effects of physical activity on the ovulatory function and the repercussions that the consequent metabolic changes determine on the ovarian function. In addition we must also consider the effects on hormones that indirectly exert effects on the hypothalamus-hypophysis-ovary axis. The use of doping substances can have an impact on the ovarian function. Finally, it is appropriate to consider an emerging aspect, the meaning of hyperandrogenism of women with polycystic ovary syndrome relative to their athletic performance.

## Abbreviations

AAS: anabolic-androgenic steroids
APED: performance-enhancing drugs
BMD: bone mineral density
DHEA: dehydroepiandrosterone
FSH: follicle-stimulating hormone
fT: free testosterone
GH: growth hormone
GnRH: gonadotropin-releasing hormone
H-OAM: hyperandrogenic oligomenorrheic/amenorrheic
HPO: hypothalamic-pituitary-ovarian
LBM: lean body mass
LH: luteinizing hormone
N-OAM: normoandrogenic oligomenorrheic/amenorrheic
NPY: neuropeptide Y
PCOS: polycystic ovarian syndrome
PRL: prolactin
RM: regularly menstruating
T: testosterone
T: testosterone
